# On Chinese species of *Dianous* group I (Coleoptera, Staphylinidae, Steninae)

**DOI:** 10.3897/zookeys.111.1431

**Published:** 2011-06-22

**Authors:** Liang Tang, Li-Zhen Li, Guang-Hong Cao

**Affiliations:** 1 Department of Biology, Shanghai Normal University, 100 Guilin Road, 1st Educational Building 323 Room, Shanghai, 200234 P. R. China; 2 Administration of Nabanhe River Watershed National Nature Reserve, Jinghong 666100, China

**Keywords:** Coleoptera, Staphylinidae, *Dianous*, Group I, China

## Abstract

Chinese species of *Dianous* group I are studied and three new species are described: *Dianous fengtingae* **sp. n.** from Hainan Province, *Dianous zhujianqingi* **sp. n.** from Jiangxi and Guizhou Province, and *Dianous huanghaoi* **sp. n.** from Yunnan Province. *Dianous shan* Rougemont and *Dianous viridicupreus* Rougemont are discovered from China for the first time. Their diagnostic characters are illustrated and a key to Chinese species of *Dianous* group I is provided.

## Introduction

The members of *Dianous* group I have large eyes and simple tarsi, and therefore were regarded as *Stenus* by earlier entomologists. In 1981 Puthz made a systematic comparison between these two genera and revealed that this group without protrudable labium surely belonged to *Dianous*. To distinguished it from other members of *Dianous*, the following characters can be used: eyes large, usually without temples; tarsi simple, without tarsal shoes; frons  with median portion not elevated.

Up to the present, 59 species of *Dianous* group I have been described, which account for nearly 30 percent of the genus. All of the species are distributed in the Oriental region and seem to be rare. In Chinese fauna, only four species were previously reported by Puthz (2000): *Dianous yao* Rougemont, 1981 from Guizhou and *Dianous tonkinensis* (Puthz), 1968 from Yunnan, Puthz (2001): *Dianous limitaneus* Puthz, 2001 from Yunnan, Shi and Zhou (2010): *Dianous viriditinctus* (Champion), 1920 from Xizang. In this paper, we complement the list with several new records and new species based on material from South China.

## Material and methods

Specimens examined in this paper were all collected near streams through forests and killed with ethyl acetate. For examination of male genitalia, the last three abdominal segments were detached from the body after softening in hot water. The aedeagus together with other dissected pieces were mounted in Euparal (Chroma Geselschaft Schmidt, Koengen, Germany) on plastic slides. Photos of sexual characters were taken with Cannon G7 attached to Olympus SZX 16 stereoscope; habitus photos were taken with a Cannon macro photo lens MP-E 65mm attached to Cannon EOS40D camera.

The type specimens treated in this study are deposited in the following public and private collections:

SHNUDepartment of Biology, Shanghai Normal University, P. R. China

cPutprivate collection of V. Puthz, Schlitz, Germany

cRouprivate collection of G.-M. de Rougemont, London, England

The measurements of proportions are abbreviated as follows:

BLbody length, measured from the anterior margin of the clypeus to the posterior margin of 10th abdominal tergite

FLforebody length, measured from the anterior margin of the clypeus to the apex of the elytra (apicolateral angle)

HWwidth of head including eyes

PWwidth of pronotum

EWwidth of elytra

PLlength of pronotum

ELlength of elytra, measured from humeral angle

## Results

### Key to Chinese species of *Dianous* group I

**Table:** 

1	Pronotum bicolorous with golden bands along the anterior and posterior margins	2
–	Pronotum unicolorous	3
2	Head distinctly broader than elytra; femora bicolorous. Habitus (Figs 13, 14), aedeagus (Fig. 3 in Rougemont 1985), female sexual characters (Figs 63–65). BL: 4.8–5.0 mm	*Dianous viridicupreus* Rougemont China (Xizang), Nepal
–	Head narrower than elytra; femora unicolorous. Habitus (Figs 17), sexual characters (Figs 6–10 in Shi & Zhou 2010). BL: 4.3–5.3 mm	*Dianous viriditinctus* (Champion) China (Xizang), India, Nepal, Bhutan
3	Body, at least head and pronotum, metallic green or golden green	4
–	Body, at least head and pronotum, metallic blue or black with plumbeous lustre	6
4	Elytra relatively narrow, head about as broad as elytra; punctures on head and pronotum moderate in size and distinctly separated, interstices can be as broad as half the diameter of a puncture. Habitus (Figs 5, 6), sexual characters (Figs 36–44). BL: 4.5 mm	*Dianous shan* Rougemont China (Yunnan), Myanmar, Thailand
–	Elytra relatively broad, head distinctly narrower than elytra; punctures on head and pronotum very coarse and very dense, interstices narrow and sharp	5
5	Frons between eyes sharply inclined inward forming a deep and broad concavity; punctures on elytra mostly distinctly delimited; paratergites of abdominal tergite 4 broad, slightly declivous. Habitus (Figs 15), aedeagus (Figs 2 in Rougemont, 1981a ). BL: 4.0–5.2 mm	*Dianous yao* Rougemont China (Guizhou), Myanmar, Thailand
–	Frons between eyes gently inclined inward forming a shallow and broad concavity, traces of two lateral longitudinal furrows can be recognized at posterior portion of the concavity; punctures on elytra mostly transversely or diagonally confluent; paratergites of abdominal tergite 4 narrow, slightly reflexed. Habitus (Figs 16), male unknown. BL: 4.5–5.2mm	*Dianous limitaneus* Puthz China (Yunnan)
6	Forebody distinctly metallic blue; femora bicolorous	7
–	Forebody black with plumbeous lustre, sometimes elytra with brassy reflection; femora unicolor	8
7	punctures on frons deep and dense; posterior half of elytra with vorticose sculpture. Habitus (Figs 11, 12), sexual characters (Figs 54–62). BL: 4.6–5.0 mm	*Dianous huanghaoi* sp. n.China (Yunnan)
–	punctures on frons shallow and sparse; posterior half of elytra with transverse sculpture. Habitus (Figs 7–10), sexual characters (Figs 45–53). BL: 3.7–4.4 mm	*Dianous zhujianqingi* sp. n. China (Jiangxi, Guizhou)
8	Punctation of pronotum and elytra coarser and less confluent; posteromedian part of 7th male sternite (Fig. 29) flattened, without keels. Habitus (Figs 3, 4), sexual characters (Figs 27–35). BL: 4.5–4.9 mm	*Dianous fengtingae* sp. n. China (Hainan)
–	Punctation of pronotum and elytra smaller and confluent; posteromedian part of 7th male sternite (Fig. 20) with an impression limited by raised keels laterally, and two acute backward projections at its posterior margin. Habitus (Figs 1, 2), sexual characters (Figs 18–26). BL: 4.4–5.9 mm	*Dianous tonkinensis* (Puthz) China (Yunnan, Hunan), Vietnam, Thailand, Borneo, Indonesia

#### Dianous tonkinensis

(Puthz), 1968

http://species-id.net/wiki/Dianous_tonkinensis

Figs 1, 2 18–26

Stenus tonkinensis Puthz 1968: 447; 1973: 41.
Dianous tonkinensis ; Puthz 1981a: 2; Rougemont 1981b: 359; Puthz 1981b: 101, 102; Rougemont 1984: 228; Puthz 2000: 501.

##### Material examined.

**CHINA: Yunnan:** male, Nabanhe N. R., Mandian, 12.I.2004, Li Li-Zhen & Tang Liang leg. (SHNU); male and female, Nabanhe N. R., Nabancun, N22°10.032, E100°39.359, alt. 720m, 6.V.2009, Hu Jia-Yao & Yin Zi-Wei leg. (SHNU); **Hunan:** male, Wufeng Town, Houhe N. R., 20. IX.2003, Ohbayashi Nobuo leg. (SHNU)

**Figures 1–6.**
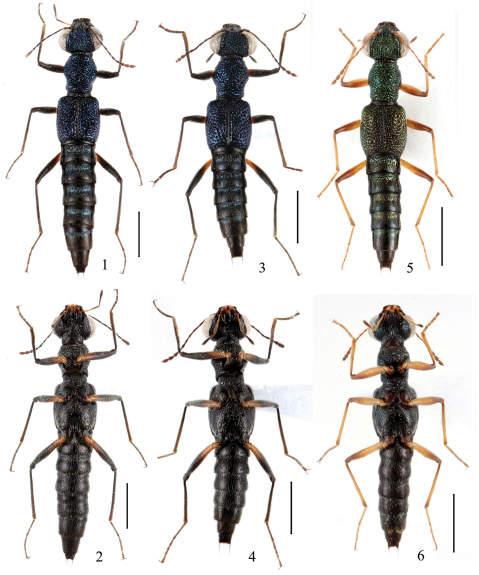
Adult habitus of *Dianous*. **1, 2** *Dianous tonkinensis* **3, 4** *Dianous fengtingae* **5, 6** *Dianous shan*. Scales = 1 mm.

##### Distribution.

China (Yunnan, Hunan), Vietnam, Thailand, Borneo, Indonesia.

#### Dianous fengtingae

Tang et Li sp. n.

urn:lsid:zoobank.org:act:54B5B60C-2D53-41A9-9B0B-0439F3466B20

http://species-id.net/wiki/Dianous_fengtingae

Figs 3, 4 27–35 

##### Type material.

**Holotype. China: Hainan:** male, glued on a card with labels as follows: “China: Hainan Prov., Ledong County, Jianfengling N. R., alt. 900m, 16.IV.2010, Feng & Yuan leg.” “Holotype / *Dianous fengtingae* / Tang & Li” [red handwritten label] (SHNU). **Paratypes.** male and 4 females, same data as for the holotype. (SHNU); female, Changjiang County, Bawangling, alt. 1000m, 14.XI.2006, Li Li-Zhen leg. (SHNU); 4 females, Changjiang County, Bawangling, alt. 450–650m, 13.IV.2010, Zhu Jian-Qing leg. (female in cPut, female in cRou, rest in SHNU).

##### Description.

Body entirely black, head, pronotum, elytra and basal abdominal tergites with a blue metallic lustre. Antennae blackish brown, with club segments lighter. First two segments of maxillary palpi brownish yellow, last segment brown. Legs blackish with tibiae and tarsi slightly lighter, femora yellowish in basal third.

BL: 4.5–4.9mm; FL: 2.5–2.6 mm.

Proportions of holotype: HW: 63.5, PW: 47, PL: 53, EW: 61, EL: 67.

Head 1.04 times as wide as elytra; interocular area gently inclined inward forming a shallow and broad concavity; punctures round, distinctly delimited, slightly larger on median area than near dorsal margins of eyes, diameter of large punctures about as wide as widest cross section of 2nd antennal segment, interstices smooth, mostly smaller than half diameter of punctures. Antennae when reflexed exceeding posterior margin of pronotum;  length of segments from base to apex: 10.0: 7.0: 17.5: 11.0: 9.0: 8.0: 8.0: 7.0: 7.0: 8.0: 10.0.

Pronotum 1.28 times as long as wide, widest slightly before middle and constricted at base; punctures round,  partially slightly confluent, distinctly larger than those on frons, interstices smooth, mostly smaller than half diameter of punctures.

Elytra nearly rectangular; punctation on average slightly coarser than that of pronotum, punctures on humeral area mostly distinctly delimited, and those on inner 2/3 portion of elytra (especially those on posterior half) obliquely confluent, interstices similar to those on pronotum.

Length of metatarsi from base to apex: 11.5: 8.5: 5.5: 3.5: 10.5.

Abdomen subcylindrical; 3rd to 6th segments with broad and densely punctate paratergites, paratergites of tergite 4 narrower than greatest width of hind tibia; 7th tergite with an apical membranous fringe; punctures on 3rd tergite as large as one eye facet, interstices smooth.

Male. Seventh sternite (Fig. 29) with a distinct posteriomedian emargination, 8th sternite (Fig. 30) with a broad triangular emargination posteromedially; 9th sternite (Fig. 31) with the apicolateral portion serrate, posterior margin slightly emarginate; 10th tergite (Fig. 32) with the posterior margin broadly rounded. Median lobe of aedeagus (Fig. 18) with an acutely pointed and setose apex (Fig. 19), parameres extending far beyond the apex of median lobe.

Female. Sternite 8 (Fig. 33) pointed posteromedially; valvifer (Fig. 34) with the posterior margin serrate; 10th tergite (Fig. 35) with the posterior margin truncate.

##### Distribution.

China (Hainan).

##### Diagnosis.

The new species is similar to *Dianous tonkinensis* (Puthz, 1968) from South Asia and *Dianous lividus* (L. Benick, 1929) from Philippines and Indonesia. It may be distinguished from both by the coarser and less confluent punctation on pronotum and especially on elytra.

#### Dianous shan

Rougemont, 1981 new to China

http://species-id.net/wiki/Dianous_shan

Figs 5, 6 36–44

Dianous shan Rougemont 1981a: 328; 1983c: 18.

##### Material examined.

**CHINA: Yunnan:** male, Nabanhe N. R., Bengganglahu, 15.I.2004, Li Li-Zhen & Tang Liang leg. (SHNU); female, Nabanhe N. R., Nabancun, N22°09.305, E100°41.291, alt. 620m, 18.XI.2008, Tang Liang leg. (SHNU)

##### Distribution.

China (Yunnan), Myanmar, Thailand.

#### Dianous zhujianqingi

Tang et Li sp. n.

urn:lsid:zoobank.org:act:03F6F311-0FA5-4860-A53B-1F53A74F970A

http://species-id.net/wiki/Dianous_zhujianqingi

Figs 7–10 45–53 

##### Type material.

**Holotype. China: Jiangxi:** male, glued on a card with labels as follows:“China: Jiangxi Prov., Yushan County, Mt. Sanqingshan, alt. 1000–1200m, 16.X.2010, Peng, Zhai & Zhu leg.” “Holotype / *Dianous zhujianqingi* / Tang & Li” [red handwritten label] (SHNU). **Paratypes.** 14 males and 19 females, same data as for the holotype (1 pair in cPut, 1 pair in cRou, rest in SHNU); male and female, Sanqingshan, alt. 700–1000m, 4.V.2005, Hu Jia-Yao & Tang Liang leg. (SHNU); **Guizhou:** male and 2 females, Mt. Fanjing, 23.VII.2003, Li Li-Zhen, Hu Jia-Yao & Tang Liang leg. (SHNU)

##### Description.

Body entirely black with a faint plumbeous lustre, elytra sometimes with brassy reflection. Antennae blackish brown. Maxillary palpi with first segment yellowish, second segment light brown and last segment brown. Legs black with a brownish tint, tibiae and tarsi slightly lighter.

BL: 3.7–4.4 mm; FL: 2.1–2.3mm.

Proportions of holotype: HW: 58.0, PW: 44.5, PL: 49.0, EW: 59.0, EL: 63.5.

Head about as wide as elytra; lateral portions of front slightly rising, medial portion concave; punctures round, distinctly delimited, slightly larger on median area than near dorsal margins of eyes, diameter of large punctures about as wide as apical cross section of 3rd antennal segment, interstices smooth, smaller than or as broad as half diameter of punctures. Antennae when reflexed extending to the posterior margin of pronotum; Length of segments from base to apex: 9.0: 6.5: 9.0: 8.0: 8.0: 5.5: 8.0: 6.0: 7.0: 6.5: 9.0.

Pronotum 1.10 times as long as wide, widest slightly before middle and constricted at base; punctures partially confluent, diameter of large punctures about as wide as apical cross section of 2nd antennal segment, interstices smooth, mostly smaller than or about as broad as half diameter of punctures.

Elytra nearly rectangular; punctation similar to that of the pronotum, punctures on humeral area mostly distinctly delimited, those on medial two thirds obliquely confluent, interstices similar to those on pronotum.

Length of metatarsi from base to apex as 18.5: 8.0: 5.5: 4.0: 11.5.

Abdomen subcylindrical; 3rd to 6th segments with broad and densely punctate paratergites, paratergites of 4th tergite as broad as greatest width of hind tibia; 7th tergite with an apical membranous fringe; punctures on 3rd tergite slightly smaller than one eye facet, interstices with very indistinct microsculpture.

Male. Seventh sternite (Fig. 47) with a very shallow emargination posteromedially, 8th sternite (Fig. 48) with a triangular emargination posteromedially; 9th sternite (Fig. 49) with distinct apicolateral projections, posterior margin slightly serrate and almost straight; 10th tergite (Fig. 50) with posterior margin broadly round. Median lobe of aedeagus (Fig. 45) with a triangularly pointed and setose apex (Fig. 46), a pair of distinct expulsion hooks, parameres extending far beyond the apex of median lobe.

Female. Eighth sternite (Fig. 51) pointed posteromedially; valvifer (Fig. 52) with posterior margin finely serrate; 10th tergite (Fig. 53) with the posterior margin broadly pointed.

**Figures 7–12.**
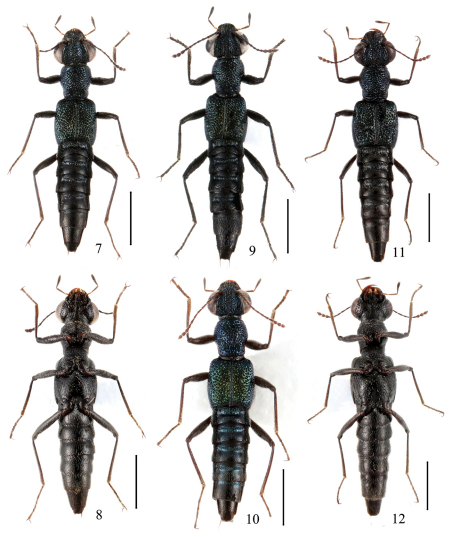
Adult habitus of *Dianous*.**7–10** *Dianous zhujianqingi* **11, 12** *Dianous huanghaoi*. Scales = 1 mm.

##### Distribution.

China (Jiangxi, Guizhou)

##### Variability.

In a few specimens the punctation of pronotum and elytra is strongly confluent as in Fig. 9. Two specimens show more a distinct brassy reflection on elytra and blue metallic reflection on basal tergites (Fig. 10).

##### Diagnosis.

The new species slightly resembles *Dianous cyaneovirens* (Cameron, 1930) from India, Nepal, Bhutan and *Dianous bracteatus* (Champion, 1920) from India, and Nepal. From both it may be easily distinguished by the faint metallic coloration (*Dianous cyaneovirens* and *Dianous bracteatus*: strongly metallic green), and from *Dianous bracteatus* also by darker legs.

#### Dianous huanghaoi

Tang et Li sp. n.

urn:lsid:zoobank.org:act:26B88EFE-CE9D-4AE4-9B4A-959AC5225830

http://species-id.net/wiki/Dianous_huanghaoi

Figs 11, 12 54–62 

##### Type material.

**Holotype. China: Yunnan:** male, glued on a card with labels as follows:“Zhonghutiao, Hutiaoxia Coun., Yunnan Prov., 24.IV.2005, Huang Hao leg.” “Holotype / *Dianous Huanghaoi* / Tang & Li” [red handwritten label] (SHNU). **Paratypes.** 2 males and 5 females, same data as for the holotype. (1 pair in cPut; rest in SHNU); 2 females, Yushuizhai, Lijiang, alt. 2600m, 14.IV.2003, stream moss, G. de Rougemont leg. (cRou)

##### Description.

Body entirely black with a faint plumbeous lustre. Antennae blackish brown, antennal club slightly lighter than preceding segments. Maxillary palpi brownish. Legs black with a brownish tint, tibiae and tarsi slightly lighter.

BL: 4.6–5.0mm; FL: 2.6–2.7mm.

Proportions of holotype: HW: 59.5, PW: 44.0, PL: 50.5, EW: 66.0, EL: 69.5.

Head 0.9 times as wide as elytra; lateral portions of frons slightly raised, median portion concave; punctures round to elliptic, distinctly delimited, slightly larger on median area than near dorsal margins of eyes, diameter of largest punctures about as wide as basal cross section of 2nd antennal segment, interstices smooth, smaller than or as broad as half diameter of punctures. Antennae when reflexed extending to the posterior margin of pronotum; Length of segments from base to apex as 9.5: 6.5: 14.5: 8.5: 7.5: 6.5: 7.0: 5.5: 6.0: 5.5: 8.0.

Pronotum 1.15 times as long as wide, widest slightly before middle and constricted at base; punctures partially confluent, similar in size to those on head, interstices similar to those on frons.

Elytra nearly rectangular; punctation similar to that of the pronotum, punctures on humeral area mostly distinctly delimited, those on posterior half of elytra strongly confluent, forming a narrowly vorticose sculpture.

Relative length of segments of hind legs from base to apex as 15.0: 8.5: 5.5: 3.5: 14.5.

Abdomen subcylindrical; 3rd to 6th segments with broad and densely punctate paratergites, paratergites on 4th segment as broad as largest width of hind tibia; 7th tergite with an apical membranous fringe; punctures on 3rd tergite distinctly smaller than eye facet, interstices smooth.

Male. Seventh sternite (Fig. 56) with a very shallow emargination posteromedially, 8th sternite (Fig. 57) with a broad emargination posteromedially; 9th sternite (Fig. 58) with distinct apicolateral projections, posterior margin finely serrate and almost straight; 10th tergite (Fig. 59) with a shallow emargination at middle of posterior margin. Median lobe of aedeagus (Fig. 54) with a triangularly pointed and setose apex (Fig. 55), parameres extending far beyond the apex of median lobe.

Female. Eighth sternite (Fig. 60) with posterior margin hardly pointed at middle; valvifer (Fig. 61) with posterior margin serrate; 10th tergite (Fig. 62) with the posterior margin rounded.

##### Distribution.

China (Yunnan).

##### Diagnosis.

The new species is similar to *Dianous carinipennis* (Bernhauer, 1914) and *Dianous nilgiriensis* Puthz, 1995, both from India. It can be distinguished from the latter two species by the less confluent punctation on pronotum and with vorticose sculpture on posterior half of elytra.

#### Dianous viridicupreus

Rougemont, 1985 new to China

http://species-id.net/wiki/Dianous_viridicupreus

Figs 13, 14 63–65

Dianous viridicupreus Rougemont 1985: 129; 1987a: 49, 50

##### Material examined.

**CHINA: Xizang:** 2 females, Nielamu County, Zhangmu Town, Lixin village, 27–28.VII.2010, alt. 2400–2600m, Zhu Jian-Qing leg.

##### Distribution.

China (Xizang), Nepal.

##### Diagnosis.

The species was originally described from Nepal and not unsurprisingly was found in China near the border.

**Figures 13–17.**
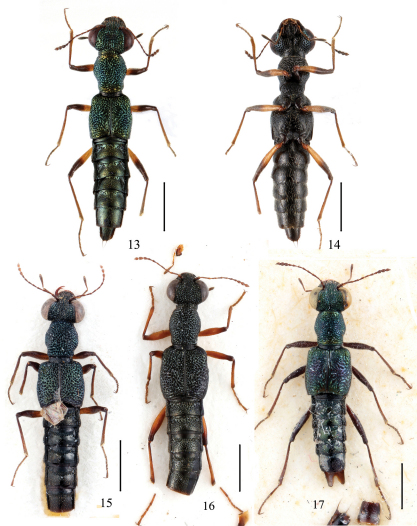
Adult habitus of *Dianous*. **13, 14** *Dianous viridicupreus* **15** *Dianous yao* **16** *Dianous limitaneus* **17** *Dianous viriditinctus*. Scales = 1 mm.

#### Dianous yao

Rougemont, 1981

http://species-id.net/wiki/Dianous_yao

Fig. 15

Dianous yao Rougemont 1981a: 330; 1981b: 359; 1983c: 18; Puthz 2000: 431, 502.

##### Distribution.

China (Guizhou), Myanmar, Thailand.

##### Diagnosis.

No Chinese material was examined by us; a photograph of a paratype (cPut) from Myanmar is provided here.

#### Dianous limitaneus

Puthz, 2001

http://species-id.net/wiki/Dianous_limitaneus

Fig. 16

Dianous limitaneus Puthz 2001: 7.

##### Material examined.

**Holotype: CHINA: Yunnan:** female, Baoshan Xian, Gongshan Mts., Lujiaba, 2400m, 10.X.1996, K. Ishii et al. leg.

##### Distribution.

China (Yunnan).

##### Diagnosis.

This species was only known from the female holotype, which is actually deposited in “the collection of the Laboratory of Entomology, Tokyo University of Agriculture”, not in “Shanghai Institute of Entomology, Academia Sinica” (Present name: Shanghai Entomology Museum, the Chinese Academy of Science) as original published paper described.

#### Dianous viriditinctus

(Champion), 1920

http://species-id.net/wiki/Dianous_viriditinctus

Fig. 17

Stenus viriditinctus Cameron 1930: 335; Abdullah and Qadri 1968: 304
Dianous viriditinctus ; Puthz 1981a: 104; Rougemont 1985: 127; Rougemont 1987: 49.

##### Distribution.

China (Xizang), India, Nepal, Bhutan.

##### Diagnosis.

No Chinese material was examined by us, and a photograph of specimen (cPut) from Nepal is provided here.

## Supplementary Material

XML Treatment for Dianous tonkinensis

XML Treatment for Dianous fengtingae

XML Treatment for Dianous shan

XML Treatment for Dianous zhujianqingi

XML Treatment for Dianous huanghaoi

XML Treatment for Dianous viridicupreus

XML Treatment for Dianous yao

XML Treatment for Dianous limitaneus

XML Treatment for Dianous viriditinctus

## Figures and Tables

**Figures 18–26.**
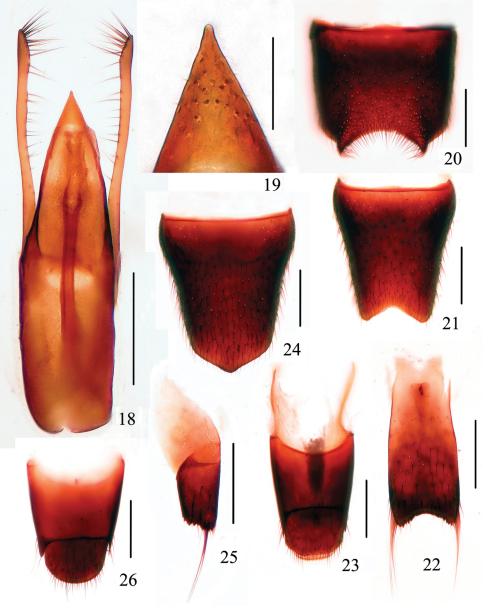
*Dianous tonkinensis*. **18** aedeagus **19** apical portion of aedeagus **20** 7th male sternite **21** 8th male sternite **22** 9th male sternite **23** 9th and 10th male tergites **24** 8th female sternite **25** valvifer **26** 9th and 10th female tergites. Scales = 0.25 mm (18, 20–26), scales = 0.05 mm (19).

**Figures 27–35.**
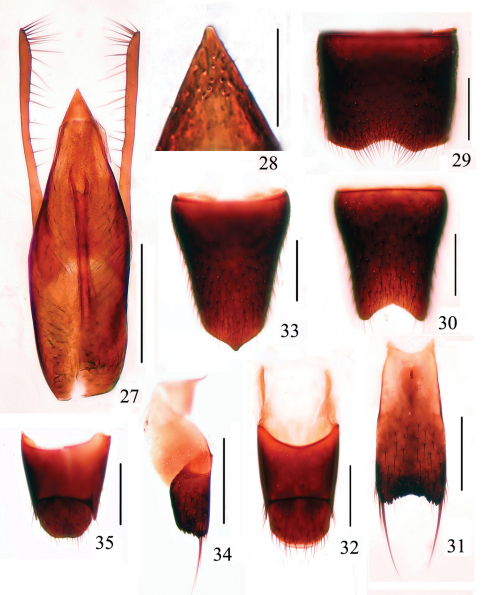
*Dianous fengtingae*. **27** aedeagus **28** apical portion of aedeagus **29** 7th male sternite **30** 8th male sternite **31** 9th male sternite **32** 9th and 10th male tergites **33** 8th female sternite **34** valvifer **35** 9th and 10th female tergites. Scales = 0.25 mm (27, 29–35), scales = 0.05 mm (28).

**Figures 36–44.**
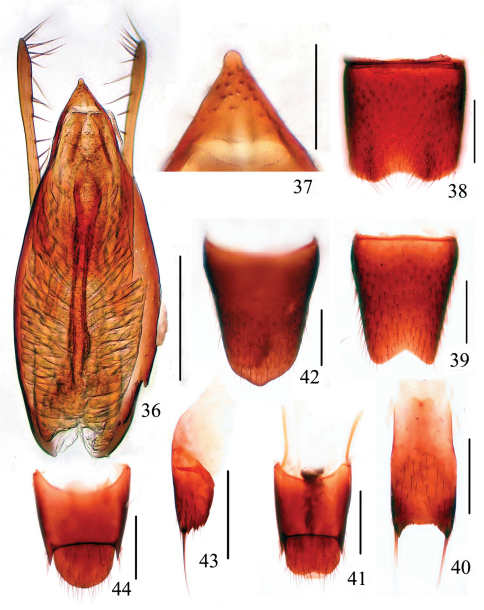
*Dianous shan*. **36** aedeagus **37** apical portion of aedeagus **38** 7th male sternite **39** 8th male sternite **40** 9th male sternite **41** 9th and 10th male tergites **42** 8th female sternite **43** valvifer **44** 9th and 10th female tergites. Scales = 0.25 mm (36, 38–44), scales = 0.05 mm (37).

**Figures 45–53.**
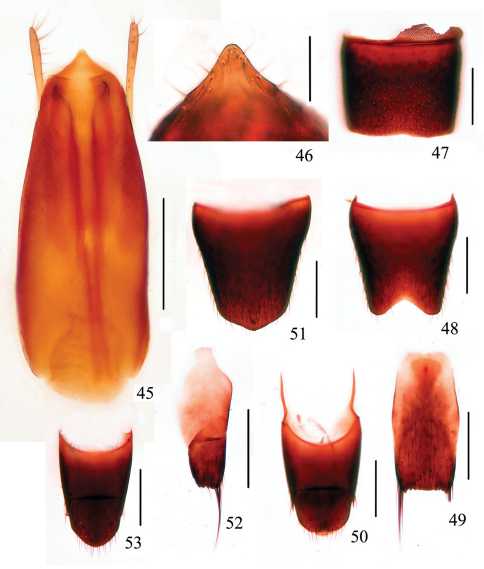
*Dianous zhujianqingi*. **45** aedeagus **46** apical portion of aedeagus **47** 7th male sternite **48** 8th male sternite **49** 9th male sternite **50** 9th and 10th male tergites **51** 8th female sternite **52** valvifer **53** 9th and 10th female tergites. Scales = 0.25 mm (45, 47–53), scales = 0.05 mm (46).

**Figures 54–62.**
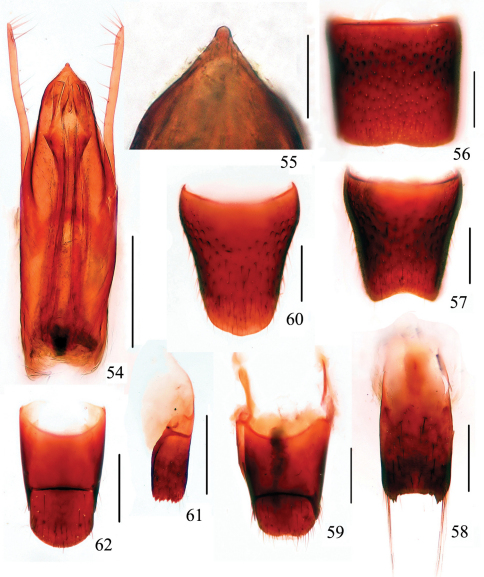
*Dianous huanghaoi*. **54** aedeagus **55** apical portion of aedeagus **56** 7th male sternite **57** 8th male sternite **58** 9th male sternite **59** 9th and 10th male tergites **60** 8th female sternite **61** valvifer **62** 9th and 10th female tergites. Scales = 0.25 mm (54, 56–62), scales = 0.05 mm (55).

**Figures 63–65.**
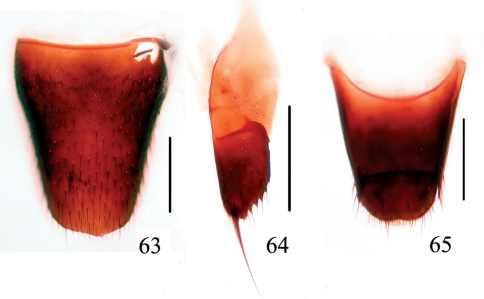
*Dianous* viridicupreus. **63** 8th female sternite **64** valvifer **65** 9th and 10th female tergites. Scales = 0.25 mm.
